# Quantitative Proteomic Profiling of Low-Dose Ionizing Radiation Effects in a Human Skin Model

**DOI:** 10.3390/proteomes2030382

**Published:** 2014-07-29

**Authors:** Shawna M. Hengel, Joshua T. Aldrich, Katrina M. Waters, Ljiljana Pasa-Tolic, David L. Stenoien

**Affiliations:** 1 Seattle Genomics, Seattle, WA 98021, USA; E-Mail: shengel@seagen.com; 2 Environmental Molecular Sciences Laboratory, Pacific Northwest National Laboratory, Richland, WA 99352, USA; E-Mails: Joshua.aldrich@pnnl.gov (J.T.A.); Ljiljana.pasatolic@pnnl.gov (L.P.-T.); 3 Fundamental and Computational Sciences Division, Pacific Northwest National Laboratory, Richland, WA 99352, USA; E-Mail: katrina.waters@pnnl.gov

**Keywords:** ionizing radiation, iTRAQ, quantitative, online 2D LC, filaggrin, skin tissue

## Abstract

To assess responses to low-dose ionizing radiation (LD-IR) exposures potentially encountered during medical diagnostic procedures, nuclear accidents or terrorist acts, a quantitative proteomic approach was used to identify changes in protein abundance in a reconstituted human skin tissue model treated with 0.1 Gy of ionizing radiation. To improve the dynamic range of the assay, subcellular fractionation was employed to remove highly abundant structural proteins and to provide insight into radiation-induced alterations in protein localization. Relative peptide quantification across cellular fractions, control and irradiated samples was performing using 8-plex iTRAQ labeling followed by online two-dimensional nano-scale liquid chromatography and high resolution MS/MS analysis. A total of 107 proteins were detected with statistically significant radiation-induced change in abundance (>1.5 fold) and/or subcellular localization compared to controls. The top biological pathways identified using bioinformatics include organ development, anatomical structure formation and the regulation of actin cytoskeleton. From the proteomic data, a change in proteolytic processing and subcellular localization of the skin barrier protein, filaggrin, was identified, and the results were confirmed by western blotting. This data indicate post-transcriptional regulation of protein abundance, localization and proteolytic processing playing an important role in regulating radiation response in human tissues.

## 1. Introduction

Humans are exposed to low levels of ionizing radiation on a daily basis due to naturally occurring background radiation, medical diagnostic procedures and occupational exposures [1,2]. There are also plausible scenarios in which large segments of the population could be exposed to both low and high doses of radiation due to terrorist acts or nuclear accidents [3]. While it is clear that large doses of ionizing radiation cause tissue and cell damage leading to disease, cancer and death, the health effects of low-dose ionizing radiation (LD-IR) are poorly understood [4]. With a high background of naturally occurring cancers, epidemiological studies of exposed individuals provide inconclusive data on the health effects of LD-IR [5], which are currently extrapolated using high dose data and a predicted linear response [6]. However, this model remains contentious in the radiation research field [5,7]. Given the increased chances for exposure to LD-IR and the public perception that any dose of radiation is harmful, it is important that the scientific community provide experimental evidence to assess potential health effects and to influence radiation regulatory policies. As a part of this, it is recognized that systems level approaches are needed to fully understand the consequences of low-dose exposures [5].

Multiple transcriptomic studies, including those on human skin *in vivo* and *in vitro* [8,9,10,11], have demonstrated that LD-IR has significant effects at the level of mRNA. However, there is scant global proteomic data available to assess LD-IR effects in a complex tissue at the protein level [12]. One study utilized an endothelial cell line exposed to a dose of 20 cGy and identified 15 altered proteins [13], while an additional study identified a handful of proteins affected by low-dose radiation (0.01 and 0.1 Gy) given alone or in conjunction with low doses of arsenic [14]. A more recent study identified multiple proteins that were persistently affected up to seven months post exposure to doses as low as 0.02 Gy in mouse heart tissue [15]. The proteins identified were linked to biological processes involving metabolism, inflammation and cytoskeletal maintenance. Our laboratory recently completed a phosphoproteomic study on a full-thickness human skin tissue model exposed to both high (2 Gy) and low (0.03 and 0.1 Gy) doses of radiation and identified multiple proteins that were affected at the post-translational level [16]. A chief finding of this study was that the skin barrier protein, filaggrin [17], exhibited altered phosphorylation following exposure to both high and low levels of ionizing radiation. This study clearly demonstrated that the reconstituted skin tissue responds to LD-IR exposure. However, many of the phosphopeptides detected were derived from a handful of abundant structural proteins, highlighting the difficulties associated with the proteomic analysis of a highly complex tissue comprised of large amounts of extracellular matrix and intermediate filament proteins.

To address challenges specifically associated with complex 3D tissues and measuring the subtle effects caused by LD-IR exposure, methods were needed to increase the dynamic range and to detect lower abundance proteins. In the work presented here, we employed a subcellular fractionation method designed to eliminate highly abundant structural proteins to address this technical limitation. As an added benefit, the analysis of individual subcellular fractions allowed for the detection of alterations in protein localization as a response to LD-IR. By combining subcellular fractionation, high resolution, 2D LC-MS/MS and iTRAQ labeling, we obtained quantitative proteomic data demonstrating statistically significant alterations in 207 proteins following exposure to ionizing radiation. Post-transcriptional regulation, including altered proteolytic processing and changes in subcellular localization, contributed to alterations in observed protein abundance.

## 2. Experimental

EpidermFT™ (MatTek, Ashland, MA, USA), full-thickness, reconstituted human skin tissues consisting of dermal and epidermal layers were sham treated or irradiated with 0.1 Gy ionizing radiation using a Pantek XRAD 320 irradiator (GE Inspection Technologies; Hurth, Germany) at a dose rate of ~0.03 Gy/min. Tissues were harvested at 8 h after radiation exposure and separated into cytoplasmic, membrane, nuclear, chromatin and cytoskeletal fractions using a subcellular fractionation kit (Thermo Fisher Scientific, Rockford, IL, USA): cytoplasmic, nuclear and chromatin fractions were selected for proteomic analysis. Proteins were acetone-precipitated to remove detergents and buffers used for the subcellular fractionation. After centrifugation, the pellets were washed with 1 mL of 90% acetone, spun down and allowed to air dry for 20 min. Proteins were then dissolved in 8 M urea, and a concentration was obtained using a standard BCA assay. Experiments were performed in biological triplicate.

To prepare for iTRAQ labeling and MS analysis, proteins were reduced, alkylated and digested with 10 mM DTT, 40 mM iodoacetamide and 1:50 protein:sequencing grade trypsin (Promega, Madison, WI, USA), respectively. Samples were then desalted using Omix C_18_ SPE tips (Varian, Santa Clara, CA, USA), quantified again with a standard BCA assay, labeled with 8-plex iTRAQ reagents (AB SCIEX, Foster City, CA, USA), according to the manufacturer’s specifications, mixed in equal amount and desalted again. For cross-injection comparison, samples consisting of equal parts of all untreated (Pool 1) or all treated samples (Pool 2) were combined, and both were added to individual reporter ion channels with individual samples. iTRAQ-labeled peptides were separated using an online, two-dimensional liquid chromatography system (2D-LC) consisting of strong cation exchange (SCX) followed by reverse phase LC. The 2D-LC system was custom built using two Agilent 1200 nanoflow pumps and one 1200 capillary pump (Agilent Technologies, Santa Clara, CA, USA), various Valco valves (Valco Instruments Co., Houston, TX, USA) and a PAL autosampler (Leap Technologies, Carrboro, NC, USA). Full automation was made possible by custom software that allows for parallel event coordination providing a near 100% MS duty cycle through the use of two trapping and analytical columns. All columns were manufactured in-house by slurry packing media into fused silica (Polymicro Technologies Inc., Phoenix, AZ, USA) using a 1-cm sol-gel frit for media retention [18]. First dimension SCX column: 5-µm PolySULFOETHYL A (PolyLC Inc., Columbia, MD, USA), 15 cm × 360 µm o.d. × 150 µm i.d. Trapping columns: 5-µm Jupiter C_18_ (Phenomenex, Torrence, CA, USA), 4 cm × 360 µm o.d. × 150 µm i.d. Second dimension reversed-phase columns: 3-µm Jupiter C_18_ (Phenomenex, Torrence, CA, USA), 35 cm × 360 µm o.d. × 75 µm i.d. The mobile phases were consisted of 0.1 mM NaH_2_PO_4_ (A) and 0.3 M NaH_2_PO_4_ (B) for the first dimension and 0.1% formic acid in water (A) and 0.1% formic acid in acetonitrile (B) for the second dimension.

LC separation was divided into 15 SCX fractions, which were separated in the reverse phase second dimension that was directly coupled to a Velos linear ion trap (LTQ)-Orbitrap mass spectrometer (Thermo Scientific, Waltham, MA, USA). The 8 most intense precursor masses in each MS scan were selected for fragmentation using both HCD and CID, for iTRAQ reporter ion abundance and peptide identification, respectively. Precursor ion MS and HCD fragment ion MS/MS scans were acquired in the Orbitrap, while CID fragment ions were measured in the LTQ. MS data were searched using SEQUEST, and peptide identifications were filtered at 1 × 10^−10^ spectral probability using MS-Generating Function (MSGF) software [19]. Reporter ion counts were determined using MS/MS Automated Selected Ion Chromatogram generator (MASiC) [20]. Results from all fractions for one multiplexed sample were concatenated. In the case of redundant peptide identifications, the event with the most abundant reporter ion count was used for quantification [21], and only unique peptide sequences were used for protein quantification. Reporter ion counts were then log_2_ normalized, and linear regression was performed to account for channel bias. Results underwent significance testing using 1-way ANOVA at the peptide level and nested ANOVA at the protein level using the in-house developed statistical suite, DanteR [22]. Multiple testing error was accounted for using the Benjamini-Hochberg *p*-value correction. The resultant peptides and proteins with *p*-values less than 0.05 were deemed significant. Functional annotation clustering and statistics were performed in the DAVID web portal [23] using gene ontology biological processes and KEGG pathway databases against the background of all identified proteins from this experiment.

## 3. Results and Discussion

**Figure 1:**
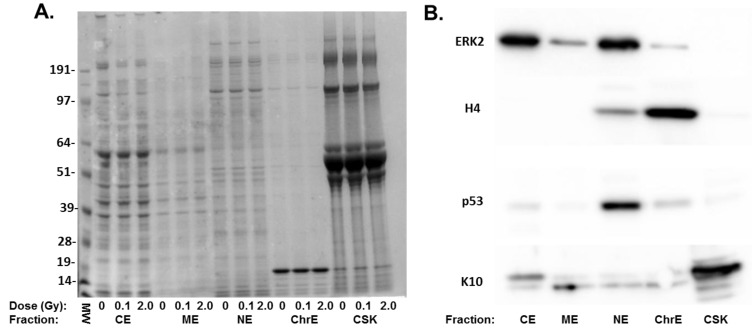
Subcellular distribution of radiation-sensitive proteins. (**A**) Coomassie blue stained gel of subcellular fractions from 0, 0.1 and 2 Gy-treated cells. Shown are the cytoplasmic extract (CE), membrane extract (ME), soluble nuclear extract (NE), chromatin extract (ChrE) and remaining cytoskeletal fraction (CSK). Molecular weight markers (MW) are shown in the first lane; (**B**) Western blots on the subcellular fractions with antibodies to ERK2, histone H4 p53 and keratin 10 (K10) to demonstrate the compartmentalization of different proteins.

Proteomic analysis of skin tissue is complicated by the highly abundant structural proteins that comprise the extracellular matrix in the dermis and cornified layer of the epidermis [16]. To improve the dynamic range of detection and to obtain quantitative data on both protein abundance and the subcellular distribution of proteins, we fractionated the tissues into five subcellular fractions representing cytoplasmic, membrane bound, nuclear, chromatin and cytoskeletal components. Aliquots of each subcellular fraction isolated from radiation-treated tissues were analyzed using gel electrophoresis to confirm that the individual fraction contained unique proteins and that the highly abundant structural proteins were present in the cytoskeletal fraction (Figure 1A). To confirm the compartmentalization of specific proteins, the five fractions were probed for proteins expected in each fraction. Western blots (Figure 1B) confirmed that each fraction showed differential enrichment for specific proteins, including ERK2 (cytoplasm and nucleus), p53 (nucleus) and histone H4 (chromatin) and keratin 10 (cytoskeletal).

**Figure 2:**
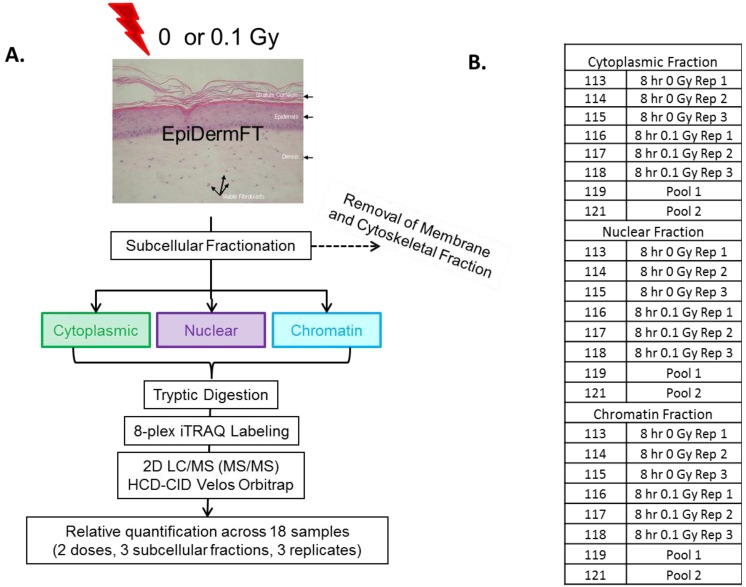
Experimental design for 8-plex iTRAQ experiment. (**A**) Reconstituted skin tissues were separated into subcellular fractions 8 h post-exposure to 0 or 0.1 Gy ionizing radiation. Each fraction was digested with trypsin, labeled with 8-plex iTRAQ reagents, recombined and analyzed using 2D LC/MS; (**B**) Each 8-plex analysis contained three biological replicates (Rep) of 0 and 0.1 Gy-treated subcellular fraction. Pooled fractions were included to facilitate comparisons across samples.

**Figure 3:**
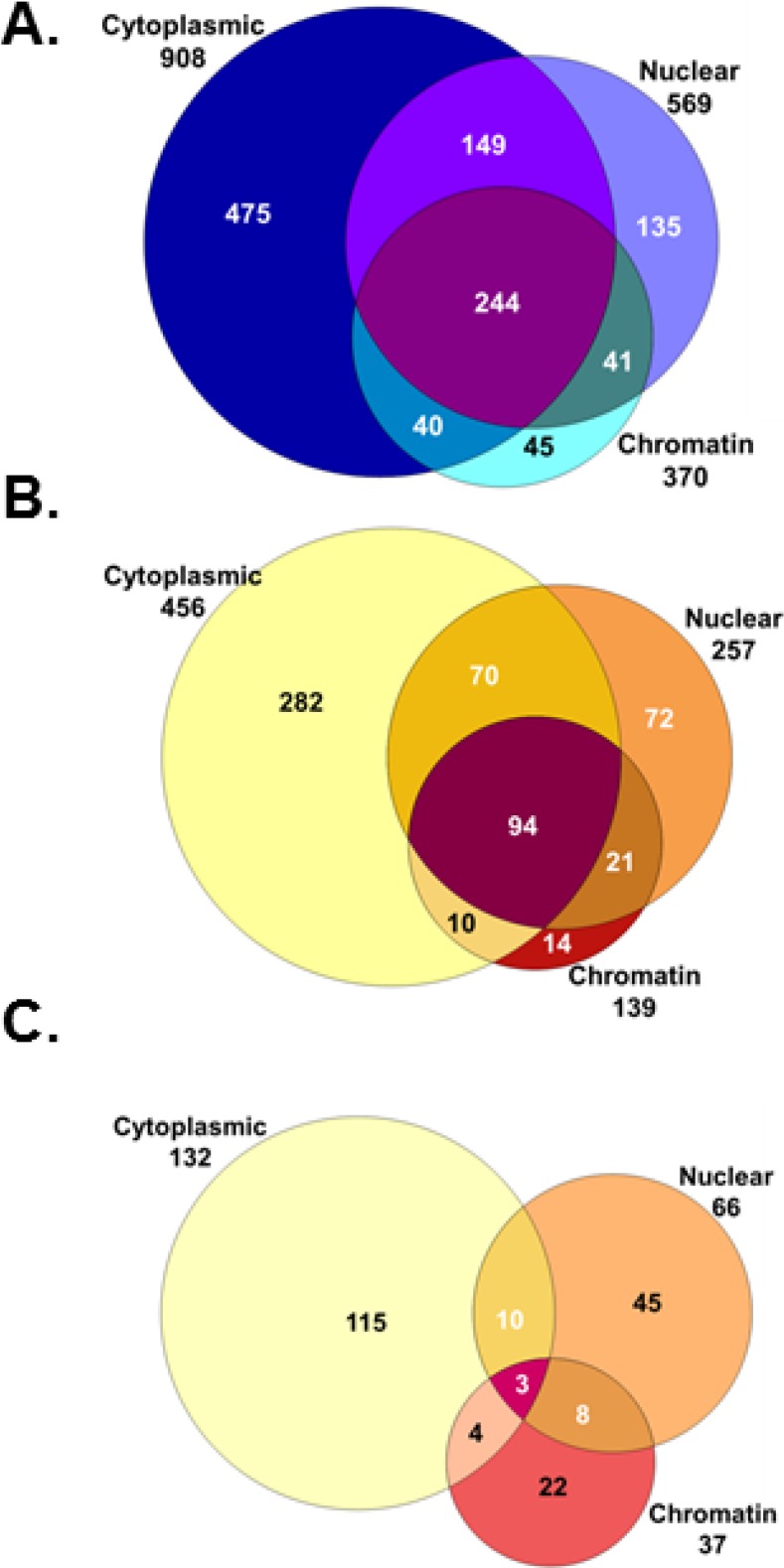
Protein identifications in each subcellular compartment. Shown are the total numbers of proteins identified (**A**), the proteins containing quantitative information (**B**) and the proteins showing alterations after radiation treatment (**C**).

In order to limit the number of tissue samples analyzed, three fractions were selected for comprehensive 2D-LC analysis. To focus on lower abundance proteins, the cytoskeletal fraction, containing highly abundant skin structural proteins, was excluded. In addition, the membrane fraction appeared to contain proteins similar to those present in the cytoskeletal fraction, but in lower abundance (Figure 1). Therefore, cytoplasmic, nuclear and chromatin fractions were chosen for a comprehensive proteomic analysis (Figure 2). An 8-h time point was selected to allow for the accumulation of protein effects occurring as a result of altered transcription and post-transcriptional mechanisms. Tissues were sham or 0.1 Gy irradiated, and subcellular fractions from three biological replicates were prepared for quantitative proteomic analysis using 8-plex iTRAQ labeling. To further increase the dynamic range of detection and reduce co-fragmentation issues (*i.e.*, adjacent, or overlapping, precursor *m*/*z* values selected for fragmentation simultaneously, which complicates quantification using iTRAQ), an online 2D-LC system consisting of strong cation exchange (SCX) followed by reverse phase (RP) chromatography was used to separate peptides. These peptides were then analyzed using a Velos linear ion trap (LTQ) Orbitrap mass spectrometer (Thermo Scientific, Waltham, MA, USA). This increased the dynamic range of detection through fractionation, while requiring minimal amounts of sample (>10 µg). A total of 1,129 proteins were identified in one or more of the subcellular fractions analyzed (Figure 3). The total proteins and their identified peptides in each subcellular compartment are listed in Supplementary Table 1. Of these, 563 proteins contained sufficient iTRAQ signal for quantitation, and 207 exhibited statistically significant changes in abundance in one or more subcellular fraction in 0.1 Gy compared to sham-treated controls (Supplementary Tables S2 and S3). Applying a fold change minimum of 1.5-fold yielded 107 radiation-affected proteins.

Among the proteins showing the greatest fold changes (Table 1 and Table 2) was the FK506 binding protein, FKBP12 (FKB1A), which underwent a 3.5-fold decrease in the chromatin fraction. FKBP12 acts as an immunosuppressant through downregulation of cytokine expression and negative regulation of mTOR signaling [24]. Decreased protein levels of FKBP12 are therefore consistent with an increased inflammatory response in skin following low-dose radiation exposure [8]. NONO and splicing factor proline/glutamine-rich (SFPQ), two DNA and RNA binding proteins with multiple nuclear functions, showed nearly identical downregulation in the nucleus (~1.7-fold) consistent with the complex formation between these two proteins [25]. Interestingly, the NONO/SFPQ complex plays an important role in DNA damage repair pathways [26,27], and the observed decrease in NONO and SFPQ after exposure to LD-IR has the potential to impair these crucial pathways.

While change in protein abundance is indicative of a biological response to LD-IR, protein abundance can potentially remain static, while subcellular translocation could indicate a significant biological event. The subcellular fractionation procedure employed prior to traditional iTRAQ sample preparation enabled the detection of changes in protein localization (Table 3). Of the 25 proteins detected to change in abundance in two or more subcellular fractions, 13 showed significant up- or down-regulation in multiple fractions, indicating an increase or decrease in overall protein abundance. Fibromodulin had the largest increase in abundance with a ~3.2-fold increase in the chromatin fraction and a smaller, but statistically significant, increase (~1.4-fold) in the nuclear fraction. While fibromodulin is a small proteoglycan typically present in the extracellular matrix [28], it has also been linked to the regulation of NF-κB signaling and apoptosis [29], indicating that it may have intracellular functions.

The remaining 12 proteins were identified with an increase in abundance in one fraction and a decrease in another, indicative of protein translocation induced by low-dose radiation exposure. Of these, annexin A2 has been independently confirmed as an LD-IR regulated protein [30], which undergoes increased translocation into the nucleus following radiation exposure [31]. The receptor for activated C-kinase 1 (RACK1; GBLP) was found in all three subcellular fractions, showing statistically significant increases in abundance in both the nuclear and chromatin compartments and a decrease in the cytoplasmic fraction. As RACK1 regulates and anchors multiple signaling molecules, including protein kinase-C isoforms [32], this potentially altered translocation could modulate protein kinase-C-related responses to ionizing radiation exposure. It is important to note that these significant biological responses would not have been identified if subcellular fractionation was not performed. This highlights the importance of looking beyond relative protein levels, as post-translational events, such as translocation, modification and protein processing, can have a large biological impact, but not necessarily affect abundance.

**Table 1:** Top downregulated proteins. The top 20 proteins with decreased abundance are shown. Fold change values are log2 scale.

Protein ID	Fraction	Change	*p*-value	Gene ID	Protein Name
FKB1A_HUMAN	Chromatin	−1.83	6.6E-05	FKBP1A	FK506 binding protein 1A, 12 kDa/FKBP12
PGS2_HUMAN	Cytoplasm	−1.67	1.7E-03	DCN	decorin
ODO2_HUMAN	Cytoplasm	−1.43	1.7E-03	DLST	dihydrolipoamide S-succinyltransferase
DBPA_HUMAN	Chromatin	−1.20	2.2E-02	CSDA	cold shock domain protein A
ATPA_HUMAN	Chromatin	−1.19	4.9E-02	ATP5A1	ATP synthase alpha subunit 1
FBLN2_HUMAN	Chromatin	−1.17	4.0E-02	FBLN2	fibulin 2
KLK10_HUMAN	Chromatin	−1.01	1.8E-02	KLK10	kallikrein-related peptidase 10
FILA_HUMAN	Nucleus	−0.99	9.8E-05	FLG	filaggrin
HUTH_HUMAN	Nucleus	−0.99	1.1E-03	HAL	histidine ammonia-lyase
HSP72_HUMAN	Cytoplasm	−0.98	1.4E-02	HSPA2	heat shock 70 kDa protein 2
SYAC_HUMAN	Cytoplasm	−0.96	2.0E-03	AARS	alanyl-tRNA synthetase
THIL_HUMAN	Nucleus	−0.93	4.1E-02	ACAT1	acetyl-CoA acetyltransferase 1
K22E_HUMAN	Nucleus	−0.91	4.5E-03	KRT2	keratin 2
SPR1B_HUMAN	Cytoplasm	−0.89	1.7E-03	SPRR1B	small proline-rich protein 1B
CAPZB_HUMAN	Cytoplasm	−0.88	1.7E-02	CAPZB	capping protein Z-line, beta
LAMC1_HUMAN	Chromatin	−0.85	3.5E-02	LAMC1	laminin, gamma 1
SFPQ_HUMAN	Nucleus	−0.85	3.3E-04	SFPQ	splicing factor proline/glutamine-rich
CYTB_HUMAN	Cytoplasm	−0.84	9.3E-04	CSTB	cystatin B (stefin B)
S10A8_HUMAN	Cytoplasm	−0.83	7.0E-07	S100A8	S100 calcium binding protein A8
MVP_HUMAN	Chromatin	−0.82	2.7E-03	MVP	major vault protein

**Figure 4:**
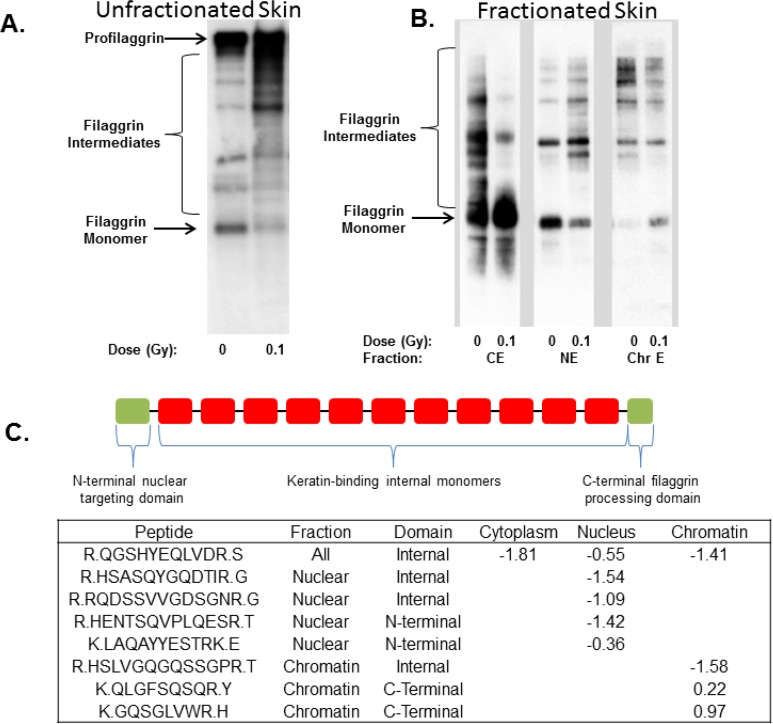
Altered processing and subcellular distribution of filaggrin intermediates. (**A**) Exposure to low-dose ionizing radiation (LD-IR) results in the accumulation of higher molecular weight filaggrin intermediates in whole tissue lysates; (**B**) Each subcellular fraction contains different filaggrin antibody-reactive bands, indicating that radiation affects both proteolytic processing and subcellular localization of specific filaggrin monomers; (**C**) Altered abundance of filaggrin peptides in each subcellular compartment.

We have previously reported that exposure of the EpidermFT skin model system to 0.1 Gy and higher doses of radiation inhibits dephosphorylation of filaggrin [16], a protein that plays an important role in the formation of the skin barrier [17,33]. Filaggrin monomers are derived from the large profilaggrin precursor through a series of regulated proteolysis steps. Since profilaggrin is restricted to the cytoskeletal fraction, our subcellular fractionation approach enabled us to examine the behaviors of filaggrin proteolytic products present in other cellular fractions. In this study, we observed an approximate two-fold decrease in the abundance of filaggrin within the nuclear fraction, confirming that low doses of radiation affect filaggrin processing. A total of five filaggrin peptides were identified showing decreased abundance in the nuclear fraction (Figure 4A). The knowledge that filaggrin undergoes proteolytic processing led us to investigate further the peptide levels across fractions. Filaggrin peptides were also detected in the cytoplasmic and chromatin fractions; however, the total protein abundance of filaggrin was not observed to change in the cytoplasm. One filaggrin peptide was detected as significantly decreasing in abundance in the cytoplasm.

**Table 2:** Upregulated proteins. The top proteins with increased abundance are shown. Fold change values are log2 scale.

Protein ID	Fraction	Change	*p*-value	Gene ID	Protein Name
FMOD_HUMAN	Chromatin	1.67	9.9E-04	FMOD	fibromodulin
TCPB_HUMAN	Cytoplasm	1.49	4.0E-03	CCT2	chaperonin containing TCP1, subunit 2
TCO1_HUMAN	Cytoplasm	1.36	1.0E-02	TCN1	transcobalamin I
ELAF_HUMAN	Chromatin	1.10	2.2E-03	PI3	peptidase inhibitor 3, skin-derived
TIMP1_HUMAN	Chromatin	1.07	3.5E-03	TIMP1	TIMP metallopeptidase inhibitor 1
MMP2_HUMAN	Chromatin	0.94	1.1E-03	MMP2	matrix metallopeptidase 2
RLA1_HUMAN	Cytoplasm	0.91	7.7E-05	RPLP1	ribosomal protein, large, P1
TSP1_HUMAN	Chromatin	0.90	4.1E-07	THBS1	thrombospondin 1
ACTN1_HUMAN	Nucleus	0.88	1.5E-05	ACTN1	actinin, alpha 1
COF1_HUMAN	Cytoplasm	0.77	7.4E-05	CFL1	cofilin 1
RL23A_HUMAN	Nucleus	0.75	3.3E-02	RPL23A	ribosomal protein L23a
CD109_HUMAN	Cytoplasm	0.74	1.3E-02	CD109	CD109 molecule
TPM3L_HUMAN	Chromatin	0.72	1.6E-02	TPM3	Tropomyosin 3
ACON_HUMAN	Chromatin	0.71	2.1E-04	ACO2	aconitase 2
PEDF_HUMAN	Chromatin	0.71	3.6E-08	SERPINF1	serpin peptidase inhibitor, clade F
CD59_HUMAN	Nucleus	0.69	6.8E-05	CD59	CD59 molecule
K1C10_HUMAN	Cytoplasm	0.69	1.5E-02	KRT10	keratin 10
GRP78_HUMAN	Chromatin	0.69	6.0E-03	HSPA5	heat shock 70 kDa protein 5
UGDH_HUMAN	Cytoplasm	0.67	4.1E-02	UGDH	UDP-glucose 6-dehydrogenase
NAGK_HUMAN	Cytoplasm	0.67	1.2E-02	NAGK	*N*-acetylglucosamine kinase

A more complex picture was present in the chromatin fraction in which four peptides were identified. Two peptides decreased in abundance, and two different peptides increased in abundance, which, when combined together, indicated that there was no net change in filaggrin abundance in the chromatin fraction. However, filaggrin is synthesized as a very large profilaggrin protein that consists of an N-terminal nuclear targeting domain, 10–12 internal keratin binding domains and a C-terminal calcium binding domain, connected by a ~19 amino acid linker region that is cleaved by calcium activated proteases to generate active filaggrin monomers [34]. Because each filaggrin monomer could be considered a unique protein, we broke down the locations of the observed peptides to the *N*-terminal nuclear targeting domain, internal domains or C-terminal domains. This separate analysis indicated that the N-terminal domain localized to the nucleus as expected [35,36] and decreased in abundance following radiation exposure. Peptides mapped to the C-terminal domain were only identified in the chromatin fraction and, in contrast to the nuclear localized N-terminal domain, exhibited increased abundance after radiation treatment. Peptides originating from the internal monomers were present in all three fractions and consistently decreased in abundance. Once peptide location within the filaggrin protein domain was considered, the peptide data as a whole pointed to alterations in protein processing and, ultimately, the subcellular location in response to LD-IR.

**Table 3:** Proteins altered in two or more subcellular fractions. Shown are the statistically significant fold changes in the 0.1 Gy-treated samples compared to controls for proteins present in more than one subcellular fraction. Proteins exhibiting potential translocation are in bold. All values are log2 scale.

Protein ID	Chromatin	Nucleus	Cytoplasm	Gene ID	Protein Name
**GBLP_HUMAN**	0.616	0.405	−0.335	GNB2L1	RACK1
MDHM_HUMAN	0.508	0.385	0.249	MDH2	Malate Dehydrogenase 2
**LAMC1_HUMAN**	−0.854	0.193	−0.455	LAMC1	Laminin gamma 1
ISK5_HUMAN		0.292	0.501	SPINK5	Serine peptidase Inhibitor
HSPB1_HUMAN		−0.475	−0.535	HSPB1	Heat shock 27 kDa protein 1
**ACTN4_HUMAN**		0.432	−0.362	ACTN4	Actinin, alpha4
**ANXA2_HUMAN**		0.350	−0.404	ANXA2	Annexin A2
**CH60_HUMAN**		0.414	−0.196	HSPD1	60 kDa chaperonin
COF1_HUMAN		0.394	0.768	CFL1	Cofilin 1
**PARK7_HUMAN**		0.296	−0.394	PARK7	Parkinson Disease 7
**HS90A_HUMAN**		0.265	−0.743	HSP90AA1	HSP90 alpha 1
**LDHA_HUMAN**		−0.440	0.358	LDHA	Lactate dehydrogenase
POSTN_HUMAN		−0.589	−0.464	POSTN	Periostin
LUM_HUMAN	0.471		0.247	LUM	Lumican
**TCPQ_HUMAN**	0.343		−0.302	CCT8	chaperonin containing TCP1, subunit 8
EMIL1_HUMAN	−0.596		−0.812	EMILIN1	Elastin microfibril interfacer 1
**CO6A2_HUMAN**	−0.727		0.336	COL6A2	Collagen, Type VI, alpha 2
FMOD_HUMAN	1.667	0.474		FMOD	Fibromodulin
GRP78_HUMAN	0.687	0.250		HSPA5	heat shock 70 kDa protein 5
MMP1_HUMAN	0.599	0.589		MMP1	Matrix metallopeptidase 1
**RINI_HUMAN**	0.544	−0.213		RNH1	Ribonuclease inhibitor 1
LEG3_HUMAN	0.485	0.171		LGALS3	Lectin 3
A2ML1_HUMAN	0.386	0.245		A2ML1	Alpha-2-macroglobulin-like 1
**DESP_HUMAN**	−0.701	0.594		DSP	Desmoplakin
DBPA_HUMAN	−1.203	−0.588		CSDA	Cold shock domain protein A

Collectively, the data presented here indicated that filaggrin proteolysis is significantly affected by LD-IR exposure. To validate this finding, we probed whole cell lysates of EpidermFT prepared 8 h after radiation exposure and observed altered filaggrin processing as evidenced by a decrease in filaggrin monomers and an increase in filaggrin intermediates (Figure 4B), consistent with our previous findings. Because the proteomic data indicated that different filaggrin monomers were altered in subcellular fractions, western blot analysis was performed on these fractions to demonstrate differential levels of filaggrin degradation products following radiation exposure in each compartment. In this case, profilaggrin was not detected in the subcellular fractions, because of partitioning within the final cytoskeletal component (data not shown); therefore, the proteomic data was most likely obtained from the fully-processed filaggrin monomers and partially-processed filaggrin intermediates, which exhibited differential solubility and subcellular fractionation. The case of filaggrin processing highlights the complexity of rolling up peptide abundance values to the protein level and suggests that studies should account for the influence of proteolytic processing and other post-translational modifications on protein quantification.

After the complete proteomic data set was evaluated for changes in protein abundance and translocation across subcellular fractions, to identify potential pathways and functions affected by LD-IR, data were analyzed using the DAVID Bioinformatics Resource [23]. The top biological pathways identified include organ development, anatomical structure formation and the KEGG pathway for the regulation of actin cytoskeleton (Supplementary Table S4), which is consistent with our previous phosphoproteomics studies [16,37,38]. Two pathway groups associated with the regulation of apoptosis were identified as being affected by LD-IR exposure. Specifically, several proteins, thrombospondin-1 (THBS1), cofilin 1 (CFL1), thioredoxin domain containing 5 (TXNDC5), heat shock 70 kD protein 5 (HSPA5) and glyoxalase 1 (GLO1), which are known to be anti-apoptotic, were present in the identified apoptotic pathways. Consistent with our findings, it has been previously demonstrated that heat shock proteins are downregulated and CFL1 is upregulated during acute dermal irritation after exposure to chemicals [39]. LD-IR exposure clearly affects proteolytic processing of filaggrin and multiple proteases, proteolytic regulators and proteasome components, as well (Supplementary Table S3).

Data were also analyzed through the use of IPA bioinformatics tools [40] to identify canonical pathways affected by exposure (Table 4). The protein ubiquitination pathway was the top pathway with 15 LD-IR affected proteins, confirming that proteolytic processing may be altered following exposure. A number of metabolic pathways, including glycolysis and the TCA cycle, were also affected and could be responsible for some of the alterations in metabolic products in these pathways observed after exposure of EpidermFT to similar doses of radiation [41]. A proteomic analysis of endothelial cells exposed to high doses of radiation (2.5 Gy) also implicated glycolysis as a potential radiation-affected pathway [42]. In addition, both enolase 1 and phosphofructokinase were upregulated in our data and in this cited work. A recent metabolomics study using very high dose exposures (8 Gy) demonstrated that TCA cycle metabolites are reduced in rat serum [43], indicating that this metabolic pathway is also a potential radiation target. Potential effects on EIF2 signaling suggest protein translation is an LD-IR target, as indicated by our published phosphoproteomic studies on skin fibroblasts [37,38]. Multiple tissue remodeling pathways, including inhibition of matrix metalloproteases and remodeling of epithelial adherens cell junctions, suggest that the LD-IR effects include potential alterations in tissue structural integrity.

**Table 4:** Top canonical pathways affected by LD-IR. The *p*-values calculated by IPA indicate the probability that a pathway is affected based on the number of affected proteins in that pathway.

Pathway	*p*-Value	Affected Proteins
Protein Ubiquitination Pathway	4.17E-7	PSMA6,PSMA1,HSPD1,HSPA5,HSPA2,PSMD8,HSPA8,PSMD11, SP90B1,PSME1,PSMA5,HSP90AA1,PSMC3,HSPB6,HSPB1
Glycolysis I	8.02E-6	ENO1,TPI1,PKM,GAPDH,PFKP
EIF2 Signaling	7.24E-5	RPL4,EIF3B,RPL5,EIF4A1,RPS8,RPL23A,RPS3,RPS23, RPLP1,RPL13
TCA Cycle II	1.44E-4	CS,ACO2,DLST,MDH2
Pentose Phosphate Pathway	2.55E-4	PGD,TKT,G6PD
Role of IL-17A in Psoriasis	4.35E-4	S100A7,S100A9,S100A8
Aldosterone Signaling in Epithelial Cells	4.75E-4	HSPA8,HSP90B1,HSP90AA1,HSPD1,HSPA5,HSPA2, HSPB6,HSPB1
Inhibition of Matrix Metalloproteases	1.15E-3	HSPG2,TIMP1,MMP2,MMP1
Tryptophan Degradation X	1.19E-3	AKR1A1,ALDH1A1,ALDH7A1
Remodeling of Epithelial Adherens Junctions	1.29E-3	ARPC2,ARPC3,VCL,ACTN4,ACTN1
Pentose Phosphate Pathway	1.38E-3	PGD,G6PD
ILK Signaling	2.05E-3	CFL1,MYL6,MYH9,VIM,ACTN4,ACTN1,DSP,NACA
Epithelial Adherens Junction Signaling	2.05E-3	MYL6,ARPC2,MYH9,ARPC3,VCL,ACTN4,ACTN1

Many of the statistically significant protein fold changes identified in this study were moderate, which could be the result of a number of factors. These include the use of a relatively mild stimulus of 0.1 Gy, which is insufficient to fully activate DNA repair and apoptotic pathways typically observed after high dose exposures. Additionally, the use of a complex tissue may dilute signals originating from only one of the stratified layers or cell types. Furthermore, technical limitations well established with the iTRAQ method can lead to a lower than expected fold change. While the trend of the fold change is typically correct when using iTRAQ [44,45], the absolute fold change can be dampened due to co-fragmentation of precursor ions. While this should have been minimized due to extensive fractionation in the study design and tight isolation windows in the MS methods, it can still occur. Another potential confounding factor that can influence protein level quantification is the presence of post-translational modifications that change in response to stimulus. If unidentified, these can alter observed peptide abundance without affecting overall protein abundance. As an example of this last point, post-translational modifications (PTMs) of vimentin, including reduced methionine oxidation in the 0.1 Gy-treated tissues (data not shown) and the potential for altered phosphorylation of some vimentin peptides [16,37,38] may have affected protein level quantification. These PTMs could also play a role in regulating vimentin protein levels.

## 4. Conclusions

In conclusion, using a highly-sensitive and quantitative proteomic approach, we identified LD-IR-responsive proteins, indicating that even low levels of ionizing radiation can impact biological functions. By pre-fractionating proteins into subcellular compartments prior to iTRAQ analyses, protein localization and potential translocation were identified in this study in addition to relative protein quantification. The altered proteins identified here exhibited little overlap with the mRNAs identified in transcriptomic experiments performed on human skin *in vivo* and *in vitro* [8,9,10,11]. This lack of overlap can be explained to some extent by the lower sensitivity of proteomics to detect and quantify all of the potentially affected proteins in the model system. However, the data presented here suggest that many radiation-induced changes in proteins occur at the post-transcriptional level, possibly through the altered regulation of protein translation, proteolysis and subcellular localization.

These findings highlight the importance of protein-based studies that add additional levels of information compared to transcriptomic analyses alone. However, as noted in several recent reviews on radiation proteomics [12,46,47], only a small number of ionizing radiation-affected proteins have been identified to date, and much more work is needed in this area to validate or challenge current paradigms relating to the health consequences of low-dose exposures. Most radiation proteomic studies performed to date have used higher doses and were done on cell monocultures, making it difficult to directly compare our data to these studies. In addition, it is likely that each study performed to date includes only a small subset of the total radiation affected proteome. With these caveats in mind, several proteins identified here and by Sriharshan *et al.* [42] exhibited similar trends in regulation at both low and high doses. These proteins include enolase 1 and phosphofructokinase, implicating the glycolytic pathway as a target of both low- and high-dose exposures. However, the handful of other proteins found in both studies showed opposite trends in regulation, opening the possibility of non-linear dose responses. The data presented here is the first step towards identifying LD-IR effects on the proteome of EpidermFT, a more complex model system than cell monocultures. More work is needed to address the effects of ionizing radiation dose, dose rate and post-exposure time on proteins in human tissues.

## Supplementary Materials

Supplementary File 1
